# A Novel Paclitaxel Conjugate with Higher Efficiency and Lower Toxicity: A New Drug Candidate for Cancer Treatment

**DOI:** 10.3390/ijms20194965

**Published:** 2019-10-08

**Authors:** Mizied Falah, Mahmoud Rayan, Anwar Rayan

**Affiliations:** 1 Institute for Medical Research, Galilee Medical Center, Nahariya 2210001, Israel; mizied.falah@gmail.com; 2 Faculty of Medicine in the Galilee, Bar-Ilan University, Safed 1311502, Israel; 3 Drug Discovery Informatics Lab, QRC—Qasemi Research Center, Al-Qasemi Academic College, Baka El-Garbiah 30100, Israel; mahmoud_ryan@hotmail.com; 4 HydroMap Limited Company, Kabul 2496300, Israel; 5 Institute of Applied Research, Galilee Society, Shefa-Amr 20200, Israel; 6 IDD Therapeutics, Wadi El-Haj 13, P.O. Box 1252, Nazareth 17111, Israel

**Keywords:** paclitaxel, lipoic acid, paclitaxel-lipoate conjugate, IDD-1040, anticancer drug, anti-tumor activity

## Abstract

Paclitaxel-lipoate (IDD-1040) is a conjugate formed by the chemical joining of the two compounds, by condensing a lipoic acid moiety to the C2′ of paclitaxel. IDD-1040 was evaluated for its anti-tumor activity and potential druggability, using an in vivo non-small-cell, lung cancer (NSCLC) xenograft mouse model. In the in vivo studies, IDD-1040 showed a maximum tolerated dose (MTD) of 250 mg/kg compared to paclitaxel (PTX), with an MTD of 20 mg/kg. Most interesting, IDD-1040 demonstrated higher anti-tumor activity, and its inhibitory activity on tumor volume (cell growth) was dose-dependent. That anti-tumor activity persisted for two weeks after cessation of IDD-1040 treatment, as opposed to PTX cessation, after which the tumor relapsed, confirming that IDD-1040 exhibits superior tumor inhibition. Similar to PTX treatment, no marked body weight decrease was observed during IDD-1040 treatment, indicating a low toxicity profile. The increase in animal body weight noted over time was due to the increasing weight of tumors, recorded in all the mouse test groups. The results also showed that mortality rate of mice was reduced by treatment with IDD-1040, more so than with PTX. Furthermore, in a preliminary study on the ex vivo distribution of IDD-1040, neutropenia was primarily concentrated in the liver 1 h after injection, and most of the drug was metabolized by the liver in 24 h. All of these results demonstrate IDD-1040’s great potential as a candidate drug for cancer treatment.

## 1. Introduction

**Figure 1:**
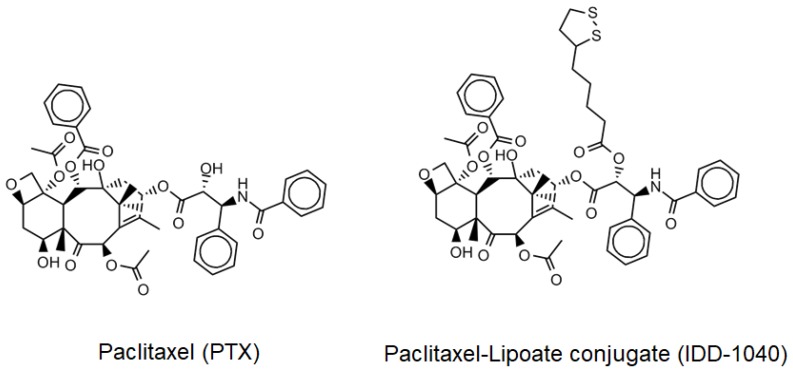
The chemical structure of paclitaxel (**left side**), and IDD-1040 (**right side**).

Paclitaxel (PTX), known by its brand name as Taxol^®^, is a diterpenoid pseudoalkaloid that consists of a taxane ring and an *N*-benzoylphenylisoserine group. It was first isolated from the tree bark of the Pacific yew, *Taxus brevifolia*, and continues to be largely used as an antitumor drug [1]. PTX’s characteristic structure is somewhat different from those of the other taxanes, due to a complex C-13 side chain attached to the taxane ring, which is important for its anti-tumor activity (Figure 1, left side). Preclinical studies have shown many promising results against many tumors, and due to its increasing demand, PTX is currently considered one of the most important anti-tumor drugs in clinical use. It is used to treat non-small cell lung cancer [2], cervical cancer [3], brain cancer [4], ovarian cancer [5], breast cancer [6], and other cancers.

The mechanism by which PTX exerts its anti-tumor activity is stabilization of the dynamic cytoskeletal structures of microtubules during the mitotic phase of cell division. In response to PTX, tumor cells, which have an accelerated cell division rate, are unable to form the highly ordered arrangement of microtubule arrays, characteristic of eukaryotic cells during cell division, so that the dynamic spindle assembly of microtubules is damaged, resulting in cell death [7,8,9]. PTX, in addition, blocks several other microtubule-based processes within the cytoplasm that are fundamental to the development and maintenance of cells [10]. It also affects major cell components involved in the processes of cell shaping and the transporting of vesicles, mitochondria, and other intracellular proteins, and it interferes with the signaling pathways that regulate processes of cell growth [9,11,12]. PTX also affects microtubule-associated proteins (MAPs) that govern the highest level of microtubule dynamics, through which chromosomes are correctly fixed, separated, and segregated during the mitotic phase of cell division [13]. Upon binding, suppressing microtubules dynamics, and preventing the progression of mitosis, PTX causes the arrest of tumor cells in the G2/M phase of the cell cycle, and thus halts the division and proliferation of these cells. Not only does PTX affect microtubules and interfere with the cell cycle progression, but it also causes changes in signaling related to apoptosis, so that cell death can occur independently of microtubule stabilization and mitotic inhibition. Moreover, it has been found that PTX is capable of binding a protein that regulates apoptosis and induces the phosphorylation of proteins—resulting in cell death [14].

Clinically, the efficacy and safety profiles of the taxanes in general, and PTX in particular, have been well established to certain extents in cancer treatment. Although PTX is still used in clinics to treat a variety of tumors [15,16,17,18,19], its use is associated with serious reactive oxygen species generation [20] and other common side effects, such as neutropenia—the most important reversible hematologic toxicity, which is dose and schedule-dependent, and neuropathy that often produces irreversible toxicity, affecting activities of daily living and overall quality of life [17]. Neuropathic symptoms are generally managed by delaying doses or reducing or discontinuing PTX administration, causing a negative impact on tumor treatment results [21]. Taxane-induced neuropathy, which develops through the inhibition of microtubule function within neurons, has been clinically studied using the drug with various modifications or with other agents or vitamin combinations [17,22]. None of these interventions has demonstrated a significant improvement in neuropathic symptoms [17].

In a recent study, the oral administration of lipoic acid in patients with painful diabetic neuropathy has been found to have a clinically significant impact on the control of neuropathy and quality of life [23]. As a strong, natural antioxidant showing extensive biological activity, lipoic acid (LA) has also been used in combination with other drugs to treat tumors and minimize the danger of neuropathy [24,25]. LA is a natural compound widely present in the body, and due to its chemical properties, it has high activity in both aqueous and lipid media. It acts both in the extracellular space and in the intracellular regions of cells, hence it has a wide range of pharmacological applications [26,27]. It is considered one of the most effective antioxidants used in daily clinical practice [28], as it exhibits protective effects against ischemia-reperfusion injuries in various organs, following the production of reactive oxygen species (ROS), which otherwise causes tissue damage [29,30]. Moreover, LA plays a protective role in diabetes and has been found to be essential in the treatment of diabetic neuropathy and cataracts [31,32]. Not only is LA effective in many trials, but it is also safe upon administration. No upper limit of LA administration has been defined in clinical use, and no adverse effects have been noticed over time at higher doses [33,34]. In regard to cancer treatment, recent studies, among many others, have confirmed that LA can inhibit cell proliferation in cervical, breast, colon, prostate, and lung cancers [24,35,36,37,38]. Most importantly, the combination of LA and PTX has shown an enhanced inhibitory effect on breast cancer cell proliferation [25]. Showing great promise, we think that this may provide a basis for qualifying LA as a chemotherapeutic drug of choice. This study aimed to explore the possible consideration of IDD-1040 as an anticancer drug candidate. IDD-1040 (Figure 1, right side) was chemically synthesized by condensing an LA moiety to paclitaxel, forming a new paclitaxel ester, and the compound was studied for its inhibitory effect on tumor cells in vitro and in vivo (using xenograft models with intravenous injection).

## 2. Results and Discussion

**Figure 2:**
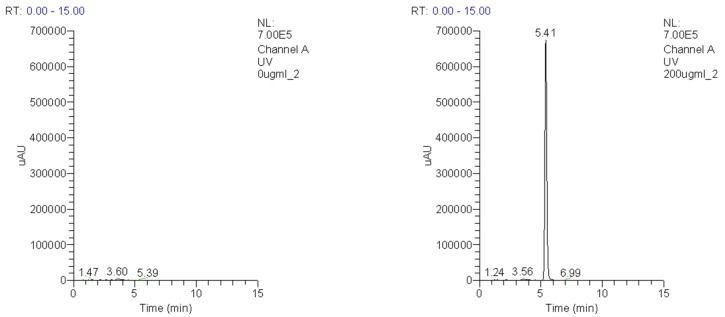
HPLC chromatograms of a blank sample (**left**) and a sample receiving 200 µg/mL IDD-1040 (**right**).

The synthesis yield of IDD-1040 product, as explained in the methods section, was 69%. The purity of the product, as assessed by HPLC, was slightly above 97%. Figure 2 depicts the typical HPLC chromatogram obtained using the isocratic mobile phase (methanol:water—82:18, *v*/*v*) at a flow rate of 1.0 mL/min with UV detection at 225 nm of a 5 μL injection. A blank sample is shown on the left and a sample receiving 200 μg/mL IDD-1040 on the right.

### 2.1. NMR Assignments

**Figure 3:**
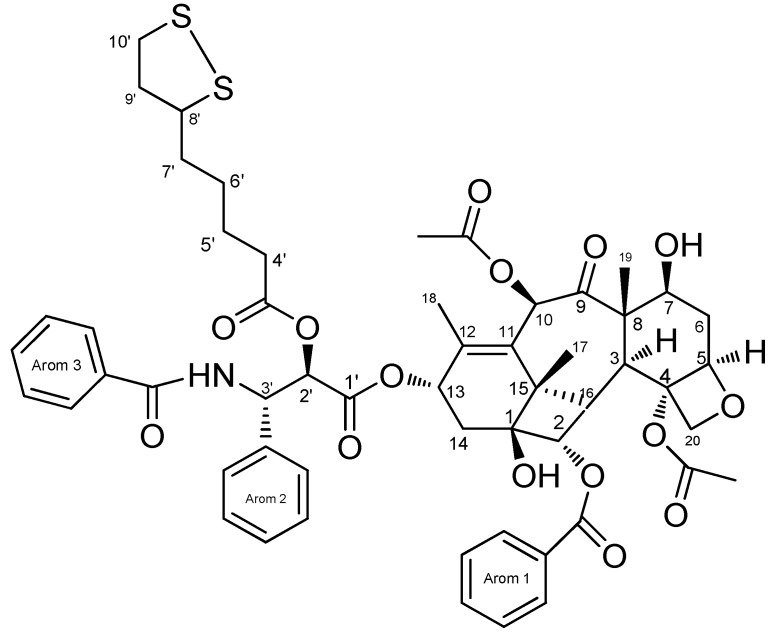
Chemical structure of the IDD-1040. Molecular formula: C_55_H_63_NO_15_S_2_.

The numbers of atoms are shown in Figure 3.

**^1^H-NMR (CDCl_3_):** 1.19 (s, CH_3_, H-16), 1.25 (m, 2H, H-5’), 1.28 (s, CH_3_, H-17), 1.28 (m, 2H, H-6’), 1.65 (m, 2H, H-5’), 1.64 (m, 4H, H4’ and H6’), 1.71 (s. CH_3_, H-18), 1.97 (m, 2H, H9’), 1.99 (ddd, 1H, H-6-β), 1.97 (s, CH_3_, H-19), 2.25 (s, 3H, OAc-10), 2.36 (m, 2H, H-4’), 2.40 (dd, 1H, H-14β), 2.44 (dd, 1H, H-14α), 2.48 (s, 3H, OAc-4), 2. 45 (m, 3H, H8’and H-10’), 2.56 (ddd, H6-α), 3.81 (d, 1H, H-3), 4.15 (d, 1H, H-2Oβ), 4.22 (d, 1H, H-2Oβ), 4.46 (ddd, 1H, H-7), 5.02 (d, 1H, H-2’), 5.31 (s, 3H, H-10), 5.58 (d, 1H, H-2), 6.24 (t, 1H), 6.33 (s, 3H, H-10), 6.90 (d, 1H, NH-3’), 7.36–7.44 (tt, 1H, p-PH-2, 5H), 7.42 (m, 5H, m-Ph3, mPh2,p-Ph2), 7.55 (m, m-Ph-3, m-Ph1, o-Ph2, 4H), 7.62 (tt, 1H, p-Ph1), 7.77 (dd, 2H, o-Ph3), 8.15 (dd, 2H, o-Ph1).

### 2.2. ^13^C-NMR (CDCl_3_)

203.5 (C-9), 172.2 (C-1), 171.1 (C=O, OAc-10), 169.9 (C=O, OAc-4), 168.1 (C=O, lipoate), 167.0 (C=O, Ph3), 167.01 (C=O, Ph1), 142.8 (C-12), 136.9 (q-Ph3), 133.8 (p-Ph1),133.1 (C11), 132.7 (qPh2), 130.01 (o-Ph1), 129.1, 128.7, 127, 127.7, 126.5 (Aromatic C), 83.2 (C-4), 81.1 (C-1), 79.1 (C-20), 76.2 (C-10), 75.4 (C-2), 75.1 (C-2’), 73.9 (C-13), 72.1 (C-7), 58.42 (C-8), 56.3 (C-8’-Lipoate), 52.67 (C-3’), 45.6 (C-3), 43.22 (C-15), 40.20 ( C-9’-Lipoate), 38.21 (C10’-Lipoate), 35.6 (C-C-14), 34.91, (C4’-Lipoate) 34.5 (C-6), 28.5 (C-17), 28.22 (C5’-Lipoate), 22.56 (4-OAcMe), 20.9 (10-OAcMe), 14.5 (C-18), 9.95 (C19).

### 2.3. Biological Study Results

The aim of this study was to define the maximum tolerated dose (MTD) for the compound IDD-1040, in order to avoid any negative parameters associated with administration, such as severe adverse behavior, weight loss > 20% compared to pre-administration, and even death. This was done to establish the form of clinical applicability, druggability, and toxicity of the new compound. The potential acute toxicity of IDD-1040 was assessed following a single, slow-bolus intravenous (IV) injection into male and female BALB/c mice.

The incidence of mortality was confined to mice injected with IDD-1040 at the highest dosages. It was found that mortality occurred during or immediately following the treatment of one out of one male mouse injected with 500 mg/kg, One male mouse injected with 350 mg/kg, and 1/1 female mouse injected with 300 mg/kg. Prior to death, the mice exhibited lateral recumbency and severe dyspnea. Clinical signs confined to mice injected with the vehicle control (Cremophor EL:ethanol absolute:physiological saline) were mainly ataxia, decreased motor activity, dyspnea, and lateral recumbency.

**Figure 4:**
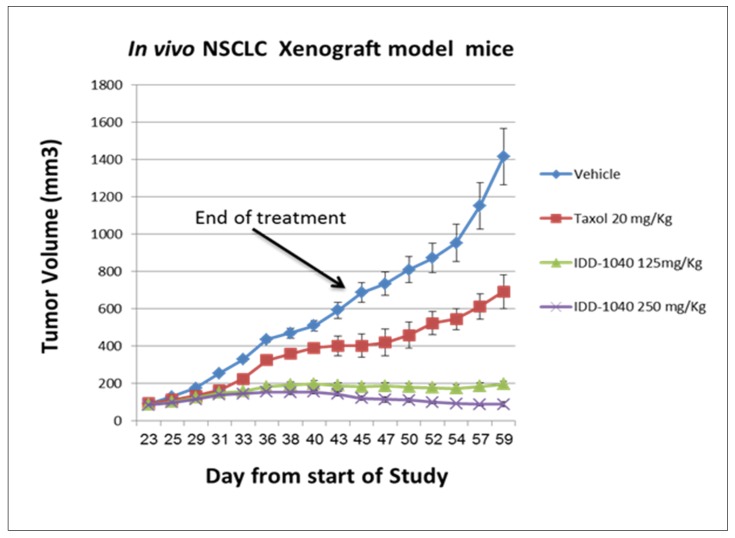
The effect of IDD-1040 on tumor volume in an in vivo non-small-cell, lung cancer (NSCLC) xenograft mouse model. The tumor size in all mice treated was measured every other day, starting three weeks after cell transplantation, and when tumor size reached about 100 mm^3^. The IV administration of vehicle, Taxol (paclitaxel) 20 mg/kg, IDD-1040 125 mg/kg, and IDD-1040 250 mg/kg was performed weekly for four weeks (on days 23, 30, 37, and 44). The administration of drug or vehicle was via IV bolus injection over one minute.

Signs were noted immediately or five minutes after post-injection and lasted up to 30 min. No clinical signs were noted in any of the surviving mice during the 14-day period, but all exhibited weight gain, which was estimated to be lower than that of the vehicle control group. Furthermore, no gross pathological findings were noted in any of the mice at the time of their scheduled necropsy, 14 days post-injection. In view of these results obtained under the conditions of this study, it may be concluded that the MTD level of IDD-1040 formulated as a stable emulsion in Cremophor EL:ethanol absolute:physiological saline following IV bolus injection over one minute into male or female BALB/c mice is 250 mg/kg. Based on the MTD concentration, IDD-1040 was further studied in an in vivo xenograft model with a human non-small-cell, lung cancer (NSCLC) A549 cell line, and the data indicate activity inhibiting tumor volume (cell growth) in a dose-dependent manner, as seen in Figure 4.

**Figure 5:**
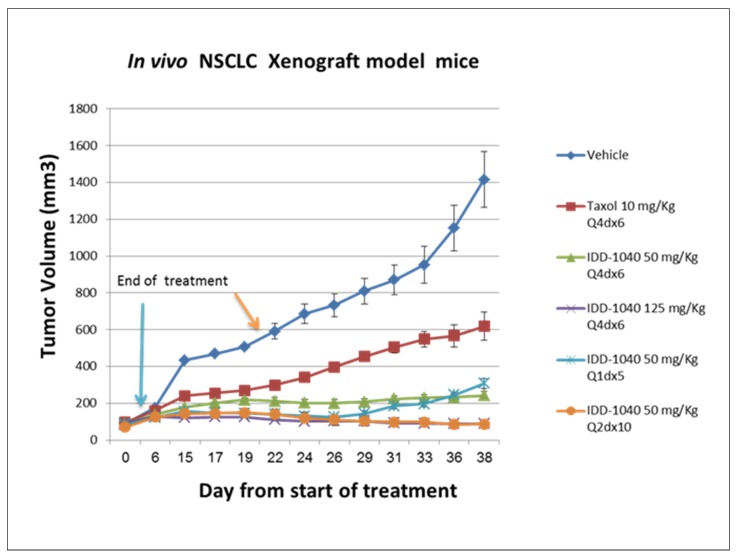
The effect of IDD-1040 on tumor volume in an in vivo NSCLC xenograft mouse model, given more frequently at lower dosages. Post-IV administration of vehicle (every 4 days for 24 days), Taxol (paclitaxel) (10 mg/kg every four days for 24 days), IDD-1040 (50 mg/kg and 125 mg/kg every four days for 24 days), and IDD-1040 (50 mg/kg once a day for five days, and every two days for 20 days).

The results clearly show that IDD-1040 profoundly inhibited tumor growth, and the results are statistically significant in comparison to the clinically approved PTX, which served as a positive control in the study. As seen, tumor growth continued after PTX cessation, confirming that IDD-1040 exhibited a superior ability to inhibit growth for two weeks after the treatment cessation. To investigate this effect in view of the MTDs of PTX (20 mg/kg) and IDD-1040 (250 mg/kg), more frequent regimens at lower doses were tested, as indicated in Figure 5.

**Figure 6:**
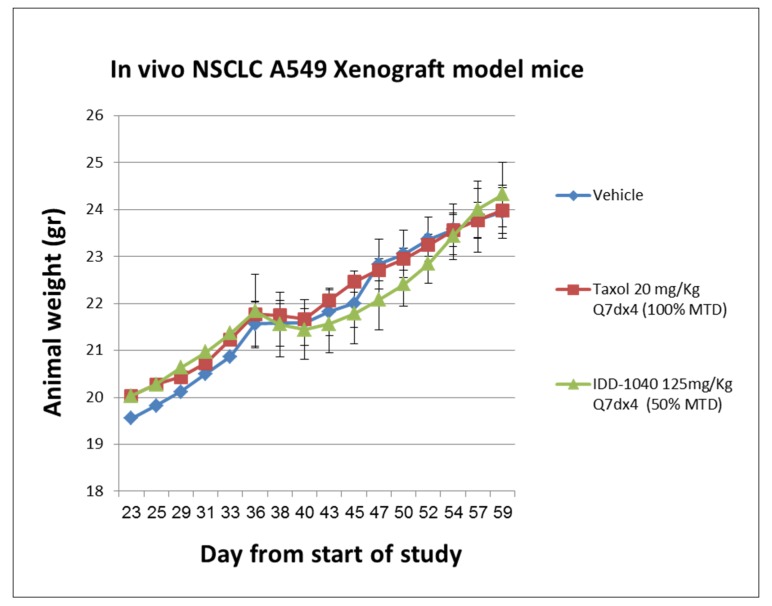
Animal body weight curves following the IV administration of IDD-1040, Taxol (paclitaxel), and vehicle in an in vivo A549 NSCLC xenograft study in mice. Animals were treated with vehicle, paclitaxel (PTX, at 100% of the maximum tolerated dose (MTD)), and IDD-1040 (at 50% of the MTD) administered weekly for four weeks.

Compared to the experiment in Figure 6, it should be noted that IDD-1040 at 20% of its MTD (a dose of 50 mg/kg) still reduced tumor growth significantly when compared to PTX at 50% of its MTD. This was seen in a variety of regimens employing dosages ranging from 20% to 100% of the MTD for IDD-1040, and 50% to 100% of the MTD for PTX. This confirms that IDD-1040 has a superior ability to inhibit tumor growth, even for another two weeks after the treatment cessation and at 20% of the MTD, which was not the case for PTX. In other words, IDD-1040, at both the MTD given weekly for four weeks and at half that dose, was more effective than PTX. Moreover, the results for PTX and IDD-1040, given more frequently, either every two or four days, show that IDD-1040 was more effective than PTX. In addition, there was a dose-response relationship for IDD-1040. All six IDD-1040 regimens that are shown in Figure 4 and Figure 5 suppressed tumor growth more than PTX, with the two regimens of 125 mg/kg every four days for 24 days (for a total of 750 mg/kg) or 50 mg/kg every other day for 20 days (for a total of 500 mg/kg) providing the best responses. Their efficacies are statistically significant when compared to untreated and paclitaxel-treated groups. The *p*-values are less than 0.01.

In order to verify the toxicity of IDD-1040 following IV administration, animal body weight and survival were monitored throughout the study. In general, a decrease in animal body weight in the range of 30% and higher is a marker for toxicity (see Figure 6).

**Figure 7:**
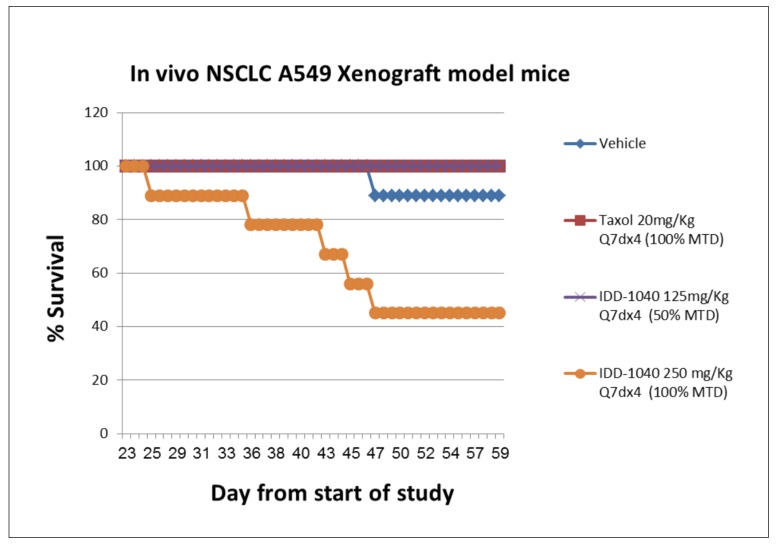
Animal survival and mortality curves following the IV administration of IDD-1040, Taxol (paclitaxel), and vehicle in an in vivo A549 NSCLC xenograft study in mice. Animals were treated with vehicle, PTX (at 100% of the MTD), IDD-1040 (at 50% of the MTD), and IDD-1040 (at 100% of the MTD), administered weekly for four weeks.

As can be seen, no marked body weight decrease was observed during the study in any of the groups; in fact, an increase in animal body weight was recorded in all groups. This observation supports the toxicity profile of IDD-1040. Tumor growth increases the weight of a mouse. Survival and mortality were also tested, and the results are shown in Figure 7.

The survival and mortality of mice was observed even in the vehicle group (11%). As expected, tumor growth reaching a level of >1400 mm^3^ may cause death in a mouse, especially in the late stage. The following regimens: IDD-1040 250 mg/kg Q7dx4 regimen (100% of the MTD, once a week for four weeks, total of four injections), IDD-1040 125 mg/kg Q4dx6 regimen (20% of the MTD, twice a week for three weeks, total of six injections), and IDD-1040 50 mg/kg Q1dx5 regimen (20% of the MTD, once a day for five days, total of five injections), caused mortality at rates of 54%, 54%, and 22% respectively, which was anticipated due to the amount administered per week to mice that were immune-deficient and bearing tumors to begin with. In the rest of the IDD-1040 treated groups (125 mg/kg Q7dx4, 50 mg/kg Q4dx6, and 50 mg/kg Q2dx10), and in the PTX-treated groups, death was not observed. Based on the survival data presented here, the administration of 150 mg/kg IDD-1040 per week in tumor-bearing mice with severe combined immunodeficiency (SCIDs) can be considered safe. Thus, a strong dependency on the dosing schedule is observed.

**Figure 8:**
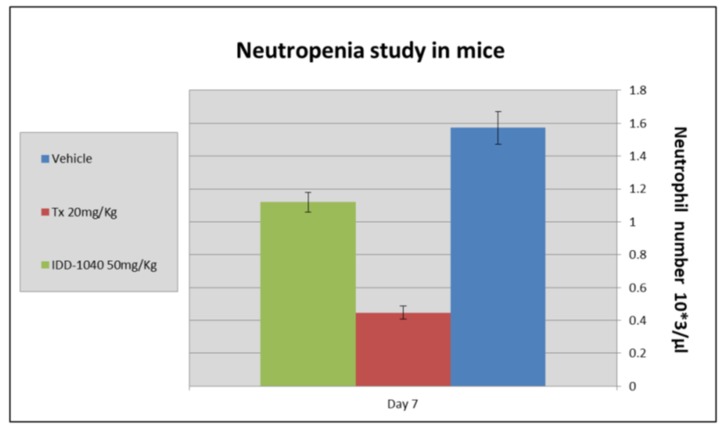
Assessment of neutropenia in mice treated with vehicle control (blue) and IDD-1040 (green) versus PTX (red).

As PTX use, like that of related anti-mitotic drugs, is generally associated with manageable toxicity, for which neutropenia is among the most common clinical side effects, the difference in toxicity between IDD-1040 and PTX was evaluated. The whole blood of the treated mice was subjected to neutrophil assessment, as indicated in results shown in Figure 8 below.

The results clearly show that IDD-1040 caused a 28.7% decrease in the neutrophil count compared to the vehicle control, while PTX caused a decrease in the neutrophil count of about 71.34% compared to the vehicle control. As can be seen, IDD-1040 induces a minimal level of neutropenia in mice compared to PTX.

Our primary goal in the current study was to introduce a safe and efficient drug candidate for cancer treatment, while minimizing the adverse side effects commonly seen in PTX-treated patients. For this purpose, a novel PTX conjugate (IDD-1040) was designed and synthesized, that attains higher efficiency and lower toxicity. To conclude, the above results clearly suggest a substantial enlargement of the therapeutic window for IDD-1040 compared to PTX. IDD-1040 was studied using an in vivo xenograft model in mice injected with NSCLC A549 cells. In that model, when both IDD-1040 and PTX were administered at the MTD, IDD-1040 was far more effective than PTX in suppressing tumor growth.

A clear dose-response curve was seen up to the MTD. In a single-dose, intravenous, non-good laboratory practice (non-GLP) toxicity study in mice, the MTD was determined to be 250 mg/kg.

**Figure 9:**
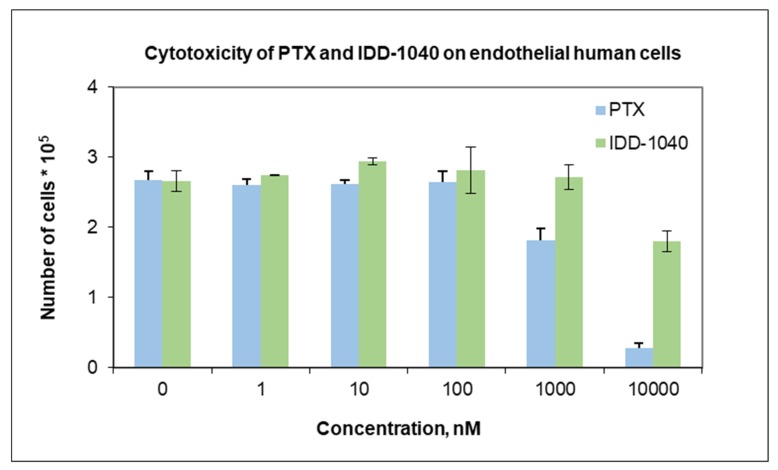
Cytotoxicity of PTX (blue) versus IDD-1040 (green).

The results from the in vivo study clearly show that IDD-1040 inhibited tumor growth more effectively than PTX, which served as a positive control in the study. Specifically, it should be noted that 20% of the MTD for IDD-1040 (50 mg/kg) reduced tumor growth significantly when compared to 50–100% of the MTD for PTX. This study confirms IDD-1040′s ability to inhibit tumor growth even two weeks after the cessation of treatment. This study in mice conceptually proves and demonstrates the efficacy of IDD-1040 against NSCLC, and thus it is worth developing. In the context of the in vitro study, IDD-1040 shows an absence of cytotoxicity in normal cells (see Figure 9), and greater in vivo efficacy in NSCLC xenograft mice (see Figure 4 and Figure 5). Thus, IDD-1040 appears to function as a pro-drug (slow release system) for PTX and this could explain the improved efficacy and lesser toxicity that are shown in the in vivo model.

Proof of concept for IDD-1040 has been established. We have presented the results of an extensive in vitro and in vivo evaluation of our lead compound, which emerges as a highly promising agent, certainly worthy of vigorous future development.

IDD-1040 induces a minimal level of neutropenia in mice compared to Paclitaxel. This result supports our expectation, based on a previously established safety profile, that side effects would be greater in mice treated with PTX than in those treated with IDD-1040.

## 3. Materials and Methods

The chemicals (paclitaxel, lipoic acid, DCC, DMAP, dry dichloromethane, ethyl acetate, and dichloromethane) were purchased from Sigma (Rehovot, Israel). Acetonitrile (ACN), HPLC-grade solvent, and water HPLC-grade were purchased from Biolab (Jerusalem, Israel).

### 3.1. Purity of IDD-1040

The purity of IDD-1040 was determined using an HPLC system (Thermo Scientific, San Jose, CA, USA), which included an Accela Pump with a degasser module, an Accela autosampler, and an Accela PDA detector. HPLC-grade acetonitrile was purchased from J. T. Baker (Phillipsburg, NJ, USA), and HPLC-grade water from Biolab Ltd. (Jerusalem, Israel). An HPLC-cartridge LiChroCART^®^ 250-4 LiChrospher^®^ 100 RP-18 (5 µm) (Merck, Roch-Ha’ayin, Israel) and a LiChroCART^®^ 4-4 LiChrospher^®^ 100 RP-18 (5 µm) guard column were used to protect the analytical column.

### 3.2. Chemical Characterization

#### 3.2.1. ^1^H-NMR and ^13^C-NMR

Proton and carbon NMR measurements of IDD-1040 were carried out on a Bruker Avance II 500 spectrometer, which was equipped with a 5-mm indirect detection probe with a Z gradient.

Positive ESI MS: The molecular mass was determined by HR-ESI–MS (high-resolution electrospray ionization mass spectrometry) in positive mode (LTQ Orbitrap XL hybrid ion trap with a high-resolution Orbitrap detection system, Thermo Scientific, Waltham, MA, USA).

#### 3.2.2. Elemental Analysis

Determination of C, H, and N was performed with a Perkin-Elmer 2400 series II Analyzer, which uses a combustion method (950–1000 °C) to convert the sample elements to simple gases. The system uses a steady-state, wave front chromatographic approach to separate the controlled gases, which are detected as a function of their thermal conductivity. High-speed microprocessor control, solid-state components, and built-in diagnostics ensure good performance and reliability. The measurement of sulfur percentage was done with the oxygen-flask combustion method and subsequent gravimetric titration and through ion chromatography analysis, using a Dionex IC system.

### 3.3. Synthesis of IDD-1040

**Figure 10:**
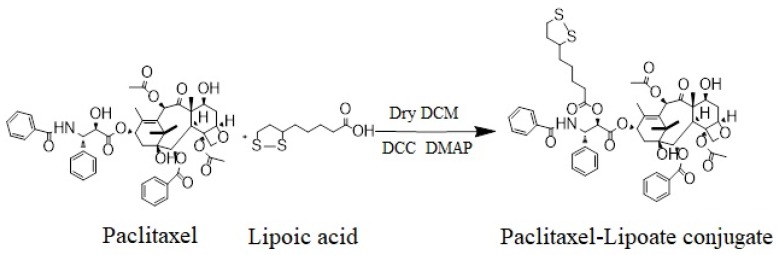
The synthesis pathway of IDD-1040.

IDD-1040 was synthesized in a one-step reaction. The condensation of PTX with LA using *N*,*N*′-dicyclohexylcarbodiimide (DCC)/4-(dimethylamino) pyridine (DMAP) as a coupling activator forms IDD-1040 (see Figure 10). To a dry DCM solution, 1.8 g (8.78 mmol) of lipoic acid and 1.8 g (8.78 mmol) of *N*,*N*′-dicyclohexylcarbodiimide (DCC) were added. The mixture was stirred for 15 min. *N*,*N*′-dicyclohexylurea (DCU) was removed by filtration, and the anhydride was added drop-wise to a solution of 5.0 g (5.85 mmol) of PTX and 700 mg (5.58 mmol) of DMAP in dry dichloromethane. The resulting mixture was stirred at room temperature with a nitrogen inlet for two hours. The organic layer was washed with 10% NaHCO_3_ once, 1% HCl twice, and finally, with brine. After drying with Na_2_SO_4_, the solution was concentrated with a vacuum. The resulting residue was separated by silica gel chromatography using DCM/ethyl acetate 7:3 as an eluent (Rf = 0.45) to yield the product (IDD-1040) as a pale-yellow solid (4.2 g, 69%). The IDD-1040 product was further dissolved in dry DCM and left at −20 °C. Traces of DCU were removed by filtration, and the solution was evaporated, yielding 4.2 g, or 69%. The purity of the product was more than 97%, as assessed by HPLC. The product was further characterized by ^1^H-NMR, ^13^C-NMR, positive ESI MS, and elemental analyses.

### 3.4. Drug Candidate Formulation for In-Vivo Experiments

IDD 1040 has lower aqueous solubility than Paclitaxel, and as a result, it was formulated as a 6 mg/mL formulation (Taxol^®^) containing 527 mg Cremophor EL and 49.7% (*v*/*v*) dehydrated alcohol. This formulation was diluted to 0.3–1.2 mg/mL before administration. The formulated solution was subjected to a stability study at RT and at 4 °C for 21 days and was found to be stable for at least 24 h under both conditions. For the toxicological and in vivo studies presented here, the formulated solution of IDD-1040 was prepared freshly before each use. As well, the control compound (PTX) and vehicle were reconstituted with a solution composed of 14% Cremophor EL, 3.5% ethanol, and 82.5% saline.

### 3.5. Animals

Two types of animal were employed in the current study: ten-week-old male and female BALB/c mice were used to carry out the experiments for MTD determination, and ten-week-old male and female NOD SCID mice were employed to investigate the drug’s effect on subcutaneous tumor growth, body weight, and neutropenia. The animals were obtained from Harlan biotech (Rehovot, Israel). They had unrestricted access to food and water, were housed in temperature and humidity-controlled rooms, and were kept on a 12-h light/dark cycle. The testing procedure was based on guidelines established by internationally accepted guidance.

To confirm whether IDD-1040 has the potential to serve as a superior anti-cancer agent, a xenograft model was employed to assess the efficacy of IDD-1040 (monotherapy) versus PTX. The study design used the following parameters: an A549 human NSCLC cell line, 7–9 SCID mice per group, and an initial tumor size of ~80–100 mm^3^. Both drugs were administered intravenously (IV) and were given in different regimens, as indicated below. There were nine test groups, with the following regimens: (1) vehicle (14% Cremophor EL, 3.5% ethanol, and 82.5% saline), (2) IDD-1040 125 mg/kg Q7dx4 (50% MTD, once a week, for a total of four injections); (3) IDD-1040 250 mg/kg Q7dx4 (100% MTD, once a week, total of four injections); (4) IDD-1040 50 mg/kg Q4dx6 (20% MTD, two times a week, total of six injections); (5) IDD-1040 125 mg/kg Q4dx6 (20% MTD, two times a week, total of six injections); (6) IDD-1040 50 mg/kg Q1dx5 (20% MTD, once a day, total of five injections); (7) IDD-1040 50 mg/kg Q2dx10 (20% MTD, three times a week, total of ten injections); (8) PTX 20 mg/kg Q7dx4 (100% MTD, once a week, total of four injections); and (9) PTX 10 mg/kg Q4dx6 (50% MTD, two times a week, total of six injections). The dosage volume in each injection was 10 mL/kg.

This study was performed following an application-form review by the National Council for Animal Experimentation and after receiving their approval (number IL-10-5-52, 11 March 2012).

### 3.6. Study Design

The procedure for establishing the MTD utilized eight groups of mice, each consisting of three males and three females. Two-hundred fifty microliters of solution (IDD-1040 solution or blank solution) were injected in a single, slow-bolus intravenous (IV) injection into male and female BALB/c mice, in the form of a stable emulsion, over one minute, at seven doses of 100, 150, 175, 250, 300, 350, and 500 mg/kg. An additional two groups of three males and three females were injected with the vehicle control item (i.e., Cremophor EL: ethanol absolute: physiological saline), and PTX 20 mg/kg, and served as negative and positive control groups, respectively. All of the surviving mice were monitored for 14 days.

### 3.7. Toxicology

A non-GLP, single-dose, intravenous toxicity study was conducted on BALB/c mice. IDD-1040 doses of Cremophor^®^ EL: ethanol absolute: physiological saline solution at 0, 100, 150, 175, 250, 300, 350, and 500 mg/kg were administered as one-minute injections, and the animals were monitored for 14 days. Groups of three of each sex were administered doses between 0 and 250 mg/kg, whereas at doses of 300, 350 and 500 mg/kg only one animal (male or female) was treated. The parameters evaluated included clinical signs, body weight, and gross pathology.

Mortality occurred at 300 mg/kg and above; all the animals in these groups died immediately post-dose. In these animals, lateral recumbency and severe dyspnea were observed. All IDD-1040-treated animals exhibited weight gain over the 14-day observation period, but the gain was lower than for the vehicle controls. No gross pathological findings were observed at necropsy in any animals. Based on these data, the single MTD was concluded to be 250 mg/kg.

### 3.8. Cell Culture

#### 3.8.1. Cancer Cells

Cell from the human, non-small-cell, lung cancer (NSCLC) cell line A549 were purchased from the American Type Culture Collection (Manassas, VA, USA) ATCC. This cell line was cultured in RPMI 1640 media (Biological Industry, Ltd., Beit Haemek, Israel), together with 1 mM of l-glutamine, 25 mM of HEPES buffer, 100 U/mL of penicillin, 100 ng/mL of streptomycin, and 10% fetal calf serum (FCS) (RPMI complete media), in an environment of 95% air and 5% CO_2_ at 37 °C.

#### 3.8.2. Normal Cells

Human umbilical vein/vascular endothelial cells (HUVECs) were purchased from the American Type Culture Collection (Manassas, VA, USA) ATCC. Endothelial cells naturally constitute the monolayer that covers the interior surface of blood vessels, and are most susceptible to circulating drugs. Thus, these cells were chosen to be investigated in vitro with IDD-1040 and PTX. A total of 70,000 cells were grown onto a 6-well-plate and after three-hour’s of cell adherence; IDD-1040 and PTX were added and the plate was incubated for 48 h in an environment of 95% air and 5% CO_2_ at 37 °C. Cells were then detached by trypsin and counted by Guava ViaCount (Guava Technologies, Millipore, Hayward, California, USA) application, a rapid and reliable method for determining cell count and viability.

### 3.9. Tumor Growth and Size Measurement in a Xenograft Model

The NOD SCID mice xenograft model was established using mice aged 8–10 weeks and weighing 20 g. The mice were studied under pathogen-free conditions within the animal house under a 12-h light/12-h dark cycle and with an ad libitum supply of standard chow often used for rodents. Cells from the lung cancer cell line A549 (NSCLC) (5 × 10^6^ cells/0.2 mL) were injected into the subcutaneous tissue of the dorsal area of each mouse, and tumors were allowed to grow to approximately 100 mm^3^ in size. Tumor volume was calculated using the following equation: tumor volume = (π/6)(width)^2^ length, where volume is measured in cubic millimeters, and width and length are measured in millimeters. The tumor size was measured three weeks after cell transplantation. The treated groups (7–9 animals per group) were mainly groups receiving vehicle, IDD-1040 (50–250 mg/kg), or Taxol (Paclitaxel) (10–20 mg/kg) according to various schedules.

### 3.10. Neutropenia

The control groups were treated with vehicle at 10 mL/kg (Group 1), with PTX at 20 mg/kg (Group 2), and with IDD-1040 at 50 mg/kg (Group 3). The administration of compounds was performed intravenously on a Q1dx5 schedule. Since both paclitaxel and IDD-1040 at the above dosages are well tolerated based on adjusted body weight (ABW, g), and showed similar efficacy against the NSCLC animal model, a daily dosing schedule was used to evaluate the toxicity potentially induced by greater compound exposure over a short period of time. Animals were monitored daily for clinical signs. On the seventh day after the initiation of therapy, all the animals were bled. Whole blood was collected into heparin-containing tubes for hematological examination and neutrophil assessment. No mortality or severe clinical signs were observed in the groups during the study.

### 3.11. Statistical Analysis

Data were analyzed using the data analysis module of excel spreadsheet software (v16.0, Microsoft, Redmond, WA, USA). Differences between the control (untreated) and treatment groups were evaluated by applying one-way analysis of variance (ANOVA). A *p*-value of less than 0.05 was considered statistically significant. The figures display the means and standard deviations.

## 4. Conclusions

Our study shows that the anti-tumor activity of IDD-1040 chemical conjugate chemotherapy in the xenograft animal model is far more favorable than that of PTX. IDD-1040 has shown a much higher MTD (>10-fold) with superior tumor inhibition. Its effect on tumor growth persists even after the cessation of treatment associated with a low-toxicity profile, as well as with a lower mortality rate in mice test groups. All of the results herein demonstrate that IDD-1040 has great potential as a drug candidate for cancer treatment, and hence, is recommended for advancement into clinical trials.
